# Adaptation and validation of a short French version of the Drive for Muscularity Scale in male athletes (DMS-FR)

**DOI:** 10.1371/journal.pone.0196608

**Published:** 2018-05-03

**Authors:** Lisa Chaba, Fabienne d’Arripe-Longueville, Vanessa Lentillon-Kaestner, Stéphanie Scoffier-Mériaux

**Affiliations:** 1 University of Teacher Education, State of Vaud (HEP-VD), Lausanne, Switzerland; 2 Université Côte d’Azur, LAMHESS, Nice, France; University of Pavia, ITALY

## Abstract

The purpose of this paper was to adapt and examine the psychometric properties of a French-language Drive for Muscularity Scale (DMS). First, a preliminary version of the scale, based on both the English-language version of the DMS and the literature on the drive for muscularity, was developed following a committee validation process. Second, the factor structure of the DMS-FR was investigated with principal component analysis (PCA) in a sample of 114 male athletes (*M*age = 23.35; *SD*age = 4.93), leading to a nine-item scale (Study 1). Third, in Study 2, the internal factor structure, temporal stability, and concurrent validity were examined through a series of structural hypothetical modelisation in a sample of 129 male athletes (*M*age = 27.03; *SD*age = 7.81). The results indicated that the scale has good psychometric properties. Specifically, the PCA, and the series of structural hypothetical modelisation, suggested two theoretical factors (i.e., Muscularity Body Dissatisfaction and Muscularity Behaviors), and more, with a bi-factor model in a SEM. The results also indicated sufficient concurrent validity with the Male Body Dissatisfaction Scale (MBDS) and adequate internal consistency (Cronbach’s alphas were .87 for the Muscularity Body Dissatisfaction subscale, and .88 for the Muscularity Behaviors subscale). The findings overall suggest good reliability and construct validity for this French version of the DMS (DMS-FR), which will be useful for future research and clinical practice in French-speaking countries.

## Introduction

Social values have changed in the past few decades, and appearance issues now affect both men and women [1]. Studies have thus begun to focus on the new male ideal body conveyed by the media and its impact on men [2,3]. This ideal consists of being strong, athletic, and thin with apparent muscular definition [4,5]. Men who think they cannot reach this ideal may develop body dissatisfaction, defined as the individual’s body-related negative self-evaluation [6]. The drive for muscularity (DM) is the perception of being underdeveloped together with the intense pursuit of increased muscle mass [7,8]. Negative outcomes of DM include: (a) eating disorders [9,10] and more specifically muscle dysmorphia, also called "reverse anorexia" [11]; (b) the use of dietary supplements [12,13] and/or doping [14,15]; (c) exercise dependence with inadequate recovery [16]; (d) the decline in social relationships to focus exclusively on strength training [17]; and (e) the risk of depression [18].

Several questionnaires have been developed to measure DM or its associated constructs. Some scales assess DM, such as the Drive for Muscularity Scale (DMS) [19], the Drive for Muscularity Attitude Questionnaire (DMAQ) [20], and the Muscle Pictorial Measure (MPM) [21]. Other questionnaires focus on body dissatisfaction, such as the Muscle Appearance Satisfaction Scale (MASS) [22] and the Male Body Dissatisfaction Scale (MBDS) [23]. Specific questionnaires also measure muscle dysmorphia, as, for example the Muscle Dysmorphia Inventory (DMI) [24]. However, to date, there is no scale in the French-language measuring the drive for muscularity.

The most extensively used questionnaire, with good levels of reliability and validity, is the DMS [19]. In the original 15-item version, the two subscales were called "Attitudes" and "Behaviors" [19]. The names were then changed to "Muscularity Attitudes and Behaviors" [25], "Muscle-oriented Body Image and Muscularity Behaviors" [26], and "Muscle-oriented Behaviors and Muscle-oriented Body Image Attitudes" [27,28]. The most frequently used names for these constructs have been "Muscle-oriented Body Image” (MBI) and Muscle-oriented Behaviors" (MB) [29–31]. The MBI subscale reflects one’s attitude toward the desire to remodel the current body shape and gain muscle mass. The MB subscale reflects the extent to which one engages in behaviors that promote a gain in muscle mass [25]. The two subscales (i.e., MBI and MB) and the global scores have demonstrated good internal consistency coefficients, test-retest reliability, and patterns of concurrent and discriminant validity [19].

In line with the recommendations of McCreary et al. [25], some studies [30] have not included item 10 (i.e. "*I think about taking anabolic steroids*") but other recent studies have found that this item loads onto the MB subscale [29].

The two-factor model of DMS scores of the parent study has been confirmed by McCreary et al. [25] and supported through CFA in many populations, including Argentinian university students [32], Spanish adolescents [26], Mexican samples [33], German weight-training men [34], male Scottish runners participating in a sporting event [35], Malaysian Malay men [27], university students from Romania [28], and Italian heterosexual and gay men [30].

Escoto et al. [33] first found a three-factor model with a split in the Behavioral dimension (i.e., attitudes, substance intake, and training adherence). The first factor had exactly the same items as in the attitudinal scale proposed by McCreary et al. [25] and later verified by McPherson et al. [35]. It should be noted that the reliability of these dimensions (i.e., attitudes, substance intake, and training adherence) was poor, and the third factor (training adherence) did not offer acceptable levels of internal consistency. McPherson et al. [35] also reported, using CFA, that the parent two-factor model of DMS scores had adequate fit. Moreover, Campana et al. [29] tested a three-factor model (muscularity concern, muscularity investment, and ambiguity of muscularity investment) but found that it had a poorer fit compared with the modified two-factor model. In this Brazilian study, the authors found that the parent two-factor model provided a better fit after elimination of three items (Items #7, 9, 10). In the Romanian translation [28], the results of PCA revealed three factors with eigenvalues > 1.0, and inspection of the scree plot suggested that there were two primary factors, with a drop-off in the third factor.

A good factorial structure was found in the study of McCreary et al. [25], revealing a single, higher-order DMS factor in both genders. Then, single higher-order DMS factor emerged in different studies [30] and some studies preferred use total scores rather than subscale scores [36]. Results about this higher-order were inconsistent: Nerini et al. [30] reported that the higher-order dimensionality had adequate fit in Italian men, while Sepúlveda et al. [26] found that a model that included the higher-order factor had poor fit in Spanish adolescents.

The DMS has been translated into several languages: Brazilian Portuguese [29], Italian [30], Spanish [26], Malay [27], German [34], and Romanian [28]. However, it has not yet been translated into French. During the translation process in other languages, researchers showed that item 1 was difficult to translate [27], item 9 included an imperial measure (pounds) not commonly used in other countries [27], and items 5, 7, and 15 came close to the cut-off for cross-loading exclusion [27]. Last, although the literature shows some variability in the factorial structure of the DMS according to social identity groups [29,30], most of the studies have supported the parent two-factor model.

Another research avenue concerns the construct validity of the scale. Despite its extensive use and the finding of Tod et al. [37] that the DMS is related to various constructs such as attitude, drive for thinness, drive for leanness, and socio-demographic variables, the first version of the DMS was not theoretically driven. Moreover, it should be noted that the MBI subscale includes items referring to attitudes, subjective norms and self-perceptions, as defined by the Theory of Planned behavior [38].

This study is not just a transcultural validation of the DMS. In link with previous transcultural studies of the DMS and observed limitations, we have chosen to adapt a short version of the questionnaire with two subscales based on the English version of the DMS. The authors of the present study have removed some items to realize validation of a questionnaire only on two concepts: body dissatisfaction and muscularity. The purpose of this study was to adapt and validate a French-language measure of the drive for muscularity to establish a more theoretically based scale for future research. In the first step, a preliminary version of the DMS was developed in French; in the second step (Study 1), the factor structure of the DMS-FR was investigated using principal component analysis; and in the third step (Study 2), the internal factor structure was examined through a series of hypothetical equation modelisation and the temporal stability and concurrent validity were assessed.

These three steps were conducted following the validation procedure of Vallerand [39] and Myers et al. [40] especially for study 2. For all studies, the ethics committees of the University of Teacher Education of the State of Vaud (Switzerland) and the University of Nice Sophia-Antipolis (France) specifically approved the protocol design and the study.

## Development of a preliminary version of the DMS-FR

The purpose of this step was to adapt a preliminary short version of the DMS in French and assess the clarity of its items.

### Methods

The development of the preliminary version followed the usual recommendations [41]. Five steps were carried out: (a) translation, (b) synthesis of the translation, (c) back-translation of the synthesis, (d) expert committee meeting, and (e) assessment of the clarity of the items. The French-language version of the DSM was called the DMS-FR.

#### Translation

The DMS scale was initially translated from English into French-language by the first and third authors, both of whom are fluent French-speakers. Two criteria guided the translation process: (a) conformity with the original questionnaire intentions and (b) clarity of the items in French.

#### Synthesis of translation

The two translated versions were compared and the two translators agreed on the preliminary version of the DSM-FR.

#### Back-translation of the synthesis

Based on this preliminary version, a back-translation was performed by a translator unaffiliated with the study and with no prior knowledge of the original instruments. The first and third authors compared the original English version of the DSM, the back-translation, and the preliminary version of the DSM-FR and worked together to ensure a clear final French-language version, equivalent to the English one in terms of semantics and concept. The two other authors did a back-translation of this final preliminary version. The back-translation was satisfactory since the back-translated questionnaire was identical to the original English version.

#### Expert committee meeting

A bilingual review committee was set up with three sport and health psychology researchers and one PhD student (the four authors). The committee first worked on the 15 basic items of the English version of the DSM and selected items which were clearly related to muscularity body dissatisfaction (e.g., "*I wish that I were more muscular*"; "*I think that my arms are not muscular enough*"; "*I think that my chest is not muscular enough*"; "*I think that my legs are not muscular enough*") and muscularity behaviors (e.g., "*I lift weights to build up muscle*"; "*I use protein or energy supplements*"; "*I drink weight gain or protein shakes*"; "*I try to consume as many calories as I can in a day*"; "*I feel guilty if I miss a weight training session*"; "*I think about taking anabolic steroids*"). Items related to other constructs such as self-efficacy (e.g., "*I think I would feel more confident if I had more muscle mass*"; "*I think that I would feel stronger if I gained a little more muscle mass*"; "*I think that I would look better if I gained 10 pounds in bulk*") or subjective norms/social approval (e. g., "*Other people think I work out with weights too often*") were removed for conceptual clarity. After the committee validation process, the final preliminary version of the DSM-FR was composed of 10 items classed into two subscales: Body Dissatisfaction Muscularity (5 items) and Muscularity Behaviors (5 items).

#### Assessment of the clarity of the items

The clarity of the items of the preliminary version of the DMS-FR was assessed by 20 males ranging in age from 21 to 41 years (*M*age = 26.43; *SDage* = 5.80) recruited through a social network. A 6-point Likert scale ranging from 1 (item not at all clear) to 6 (item quite clear) was chosen to remove the neutral option at the mid-point. Questionnaire completion was carried out under standardized conditions (i.e., isolation, paper, pencil) and did not exceed more than 10 minutes. Then, meetings were held to elicit feedback on problems understanding the questionnaire items or questions about them (e.g., understanding, meaning, relevance).

### Results

#### Clarity assessment

The descriptive statistics related to clarity assessment showed that the preliminary version of the questionnaire was very clear for the participants (*M* = 5.65; *SD* = .43). The clarity of each item was satisfactory for the two subscales of Muscularity Body Dissatisfaction and Muscularity Behaviors (i.e., 96% and 89%, respectively). A few minor changes were made on the basis of comments. The final 10 translated items of the DMS-FR are presented in Table 1.

**Table 1:** Final translated items of the DMS-FR.

Items
1—J’aimerais être plus musclé (*I wish that I were more muscular*)
2—Je fais de la musculation pour prendre de la masse musculaire (*I lift weights to build up muscle*)
3—Je prends des protéines ou des compléments énergétiques (*I use protein or energy supplements*)
4—Je bois des boissons hyperprotéinées ou aidant à la prise de masse musculaire (*I drink weight gain or protein shakes*)
5—J’essaie de consommer le plus grand nombre de calories possible par jour (*I try to consume as many calories as I can in a day*)
6—Je culpablise si je manque une séance de musculation (*I feel guilty if I miss a weight training session*)
7—J’envisage de prendre des stéroïdes anabolisants (*I think about taking anabolic steroids*)
8—Je trouve que mes bras ne sont pas assez musclés (*I think that my arms are not muscular enough*)
9—Je trouve que mon torse n’est pas assez musclé (*I think that my chest is not muscular enough*)
10—Je trouve que mes jambes ne sont pas assez musclées (*I think that my legs are not muscular enough*)

### Discussion

The aims of this first step were to adapt a preliminary short version of the DMS in French, with a focus on body dissatisfaction and muscularity constructs, and to assess the clarity of the items. Clarity assessment of the 10 items of the questionnaire provided good results.

## Study 1

The purpose of study 1 was to examine the factorial structure of the French version of the DMS (DMS-FR) using principal component analysis (PCA).

### Methods

#### Participants and procedures

The exploration of the factorial structure of the DMS-FR was conducted with 114 men, all of whom were eligible to participate in the study because they met the following criteria: (a) a minimum age of 16 years, (b) more than one hour of physical training per week, and (c) more than one year of sports experience. Participants were 18–48 years old (*M*age = 23.35; *SD*age = 4.93) and had been practicing sport from 1 to 30 hours per week for 9.76 years on average (*SD*nb of year = 5.44). All were French-speaking athletes practicing mostly bodybuilding or strength training. In this sample, 25 male athletes were not competitors and 89 male athletes competed at different levels. All participants were from France or the French-speaking part of Switzerland. A coding system was adapted to ensure no duplicate data.

The data of study 1 were collected over six months and the participants were recruited from a social network or in gyms. Written informed consent was obtained from the participants prior to participation, and our study did not include minors. Online questionnaire completion did not exceed more than 20 minutes and responding to all questions was mandatory. The participants were informed beforehand that the questionnaire was not a test (i.e., there were no right or wrong answers) and that all responses would be used for research purposes only. Participation was entirely voluntary and full confidentiality was guaranteed. The authors collected information about nationality in order to ensure that the participants were French-speakers.

#### Measures

At the beginning of the questionnaire, participants were first invited to give personal information before responding to the DMS-FR: their age and main sport (club or not; number of practice years; number of training sessions per week; competition or not; level of competition).

***Drive for Muscularity Scale (DMS-FR)***: At this stage, the DSM-FR was composed of 10 items. Participants answered each item with a Likert scale ranging from 1 (not at all) to 6 (absolutely).

#### Data analysis

***Parallel Analysis***: The factorial structure was examined by principal axis factor analysis (*Promax-type* rotation). In order to extract the most appropriate factors, parallel analysis [42] was used. In the random distribution, values lower than the factor weights were shown only for the first two factors [i.e., factor 1 (random value) = 1.52, (ACP value) = 3.88; factor 2 (random value) = 1.37, (ACP value) = 1.61]. This extraction method revealed five factors without constraint to the model. Next, the two-factor model was examined by factor analysis without additional constraint. The following items were not retained: items showing saturation coefficients above 0.40 on the two factors simultaneously, those whose saturation coefficients did not reach this value on either of the two factors, and those that did not saturate on a single factor that loaded most of the items with similar semantic contents [43]. The items were loaded onto five factors pertaining to the drive for muscularity in the following contexts: (a) body dissatisfaction and (b) muscularity.

### Results

#### Parallel analysis

Based on several indicators from this analysis, including the scree plot, eigenvalues, and proportion of variance, a two-factor solution accounting for 69.2% of the common variance was found. The first factor was labeled Muscularity Body Dissatisfaction and the second factor was labeled Muscularity Behaviors (Table 2). The factor 1 (4 items) explained 18.32% of the total variance and the factor 2 (5 items) explained 50.91% of the total variance. One item loading on the Muscularity Body Dissatisfaction factor but corresponding to the Muscularity Behaviors factor was removed (i.e., "*I lift weights to build up muscle*"). The nine remaining items all showed item-total factorial weights greater than .40. Cronbach’s alphas were .87 for the Muscularity Body Dissatisfaction subscale and .85 for the Muscularity Behaviors subscale, demonstrating high levels of internal consistency of the subscales [44].

**Table 2:** Results of the principal component analysis (PCA).

Items	Factor loading	
	Muscularity Behaviors	Muscularity Body Dissatisfaction
1—J’aimerais être plus musclé (*I wish that I were more muscular*)	.18	.80
8—Je trouve que mes bras ne sont pas assez musclés (*I think that my arms are not muscular enough*)	-.07	.85
9—Je trouve que mon torse n’est pas assez musclé (*I think that my chest is not muscular enough*)	-.19	.94
10—Je trouve que mes jambes ne sont pas assez musclées (*I think that my legs are not muscular enough*)	.10	.81
6 –Je culpabilise si je manqué une séance de musculation (*I feel guilty if I miss a weight training session)*	.49	.28
3—Je prends des protéines ou des compléments énergétiques (*I use protein or energy supplements*)	.93	-.06
4—Je bois des boissons hyperprotéinées ou aidant à la prise de masse musculaire (*I drink weight gain or protein shakes*)	.89	-.07
5—J’essaie de consommer le plus grand nombre de calories possible par jour (*I try to consume as many calories as I can in a day*)	.73	.07
7—J’envisage de prendre des stéroïdes anabolisants (*I think about taking anabolic steroids*)	.90	-.13

The factor loadings of the item removed ("*2—I lift weights to build up muscle*") were .13 (Muscularity Behaviors) and .79 (Muscularity Body Dissatisfaction).

### Discussion

The aim of study 1 was to explore the factorial structure of the DMS-FR. The PCA produced a two-factor model that replicated the original DMS subscales [19] and those of the subsequent studies [30], while providing a shorter and theoretically based version of nine items.

## Study 2

The aim of study 2 was to examine the psychometric properties of the DMS-FR by conducting a series of structural hypothetical modelisation according the four steps of Myers et al. [40]. We then examined temporal stability, internal consistency, and concurrent validity of the scale through relationships with the Male Body Dissatisfaction Scale (MBDS) [45].

### Methods

#### Participants and procedures

The 129 selected participants were 16–57 years old (*M*age = 27.03; *SD*age = 7.81) and had been practicing sport from 1 to 20 hours per week for 7.76 years on average (*SD*number of years = 6.82). The participants were French and Swiss male athletes practicing mostly bodybuilding or strength training. In this sample, 91 male athletes were not competitors and 38 male athletes were competitors at different levels. Correlations between age, sport experience and the DMS did not show significant results. When considered as covariates, the results of the exploratory structural equation modelling (ESEM) did not change. This yielded no significant effect for these two variables. This sample size was considered appropriate according to the 1:10 subjects-to-variables ratio generally adopted in the literature for CFA or SEM sample size calculations [46], and according to the strength of the factors and the items [47]. In study 1, the PCA provided two factors with 4 and 5 items with main loadings higher than .70 which could support a sample as low as 100 [48].

The procedure followed in this study was exactly the same as the study 1.

To test the temporal stability, 61 male athletes (*M*age = 25.06; *SD*age = 6.65) from the total sample were contacted four weeks after responding to the first DMS-FR and agreed to complete it a second time under the same conditions.

#### Measures

***Drive for Muscularity Scale (DMS-FR)***: At this stage, the DMS-FR consisted of nine items and two factors: Muscularity Body Dissatisfaction (MBD; 4 items) and Muscularity Behaviors (MB; 5 items). The items were answered with a Likert scale from 1 (not at all) to 6 (absolutely).

***Male Body Dissatisfaction Scale (MBDS)***: The MBDS [23] was validated in French by Rousseau et al. [45]. It is composed of two subscales: Muscularity Body Dissatisfaction (8 items) and General Body Appearance Dissatisfaction (10 items). This scale demonstrated good internal consistency: Cronbach’s alpha was .88 for Muscularity Body Dissatisfaction and .82 for General Body Appearance Dissatisfaction.

#### Data analysis

For the factorial validity, because of the significant multivariate non-normality of the data (normalized skewness: 1.0; normalized kurtosis: 10), the analysis were performed with the AMOS 7.0 [49] using the maximum-likelihood method of bootstrap. The series of structural hypothetical modelisation was conducted according to the method of Myers et al. [40]: (a) an unidimensional model; (b) a first-order model with two correlated factors; (c) a second-order hierarchical model; and (d) a confirmatory bi-factor model. The following fit indices were used: χ2; df; the Root Mean Square Error of Approximation (RMSEA), the Comparative Fit Index (CFI), and the Tucker-Lewis Index (TLI). A model in which CFI and TLI were greater than .90 [50] and RMSEA was equal to or lower than .08 and .05 [50,51] was considered satisfactory. The Akaike Information Criterion (AIC) and the Expected Cross-Validation Index (ECVI) were also analyzed. The AIC and ECVI are not normed on a zero to one scale. Reductions of their values, in comparison with other competing models, demonstrated an improved and more parsimonious fit of a model [52]. SPSS Statistics version 24 was used to examine the temporal stability and concurrent validity of the scale with Pearson correlations.

### Results

#### Confirmatory factor analysis

**Fig 1:**
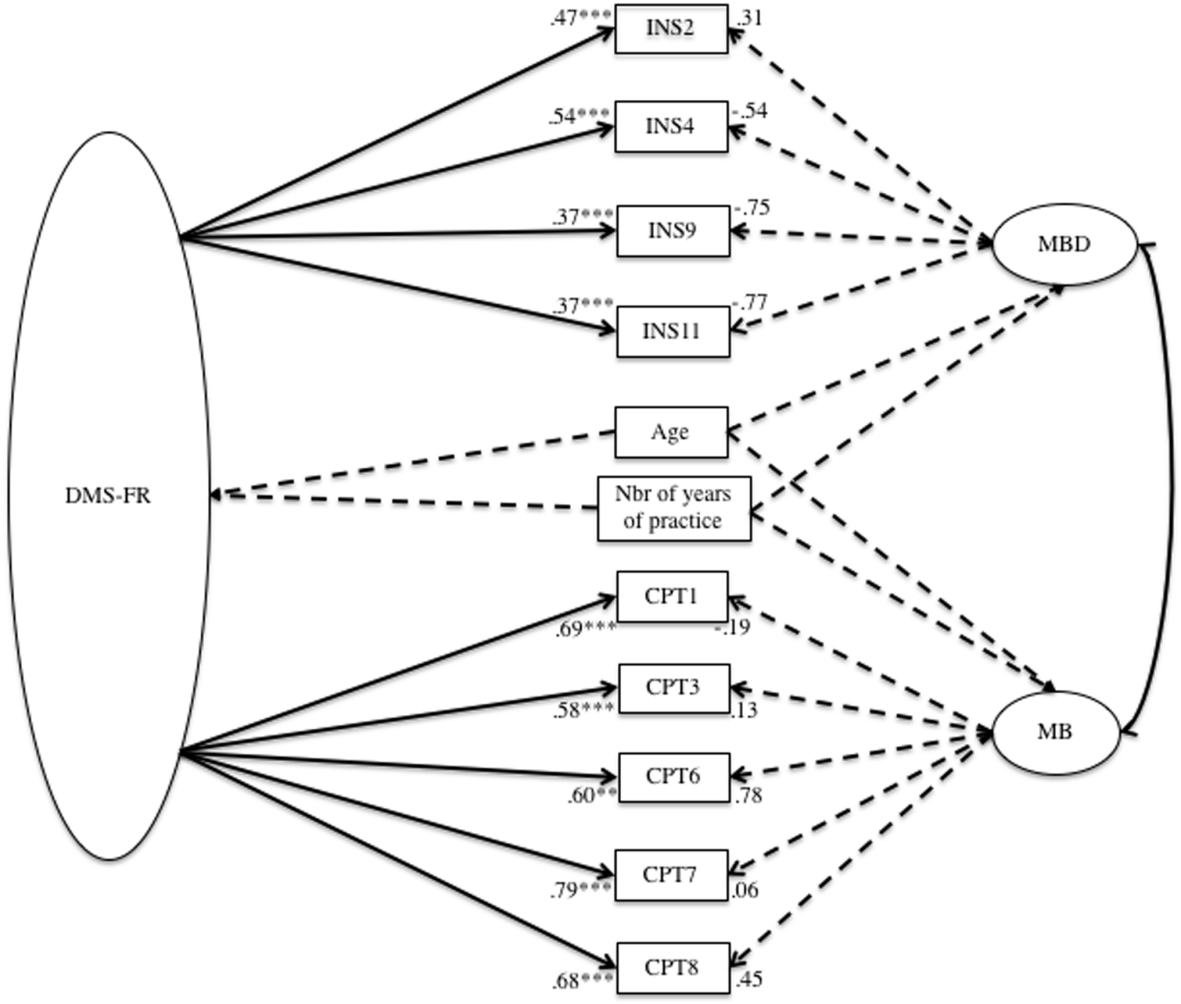
The bi-factor model with a general factor: Drive for Muscularity (DMS-FR) and two sub-factors: Muscularity Body Dissatisfaction (MBD) and Muscularity Behaviors (MB).

The results are presented in Table 3. First, the unidimensional model had shown satisfactory adjustment indices (model a). The following analysis examined a first-order model with two correlated factors (model b) and a second-order hierarchical model (model c). Finally, the analysis using a bi-factor model (model d, Fig 1) revealed that this nine-item model presented the most satisfactory adjustment indices: χ2 (28) = 28.3; *p* < .001; TLI = .99; CFI = .99; RMSEA = .01. Comparison of acceptable models (i.e., models a, b, c and d) revealed that model d provided the best goodness of fit indices, as well as the lowest ECVI and AIC indices [52]. Satisfactory internal consistencies were found: Cronbach’s alpha was .87 for the global scale, .87 for the Muscularity Body Dissatisfaction subscale and .85 for the Muscularity Behaviors subscale.

**Table 3:** Adjustment indices of the different structural equation modeling analyses.

	χ2 (df)	*p*	RMSEA	TLI	CFI	AIC	ECVI
**Model a**	46.13 (32)	< .001	.06	.95	.97	136.11	1.10
**Model b**	36.25 (34)	< .001	.02	.99	.99	122.20	0.95
**Model c**	36.61 (35)	< .001	.02	.99	.99	120.64	0.91
**Model d**	28.31 (28)	< .001	.01	.99	.99	104.32	0.83

Model a: unidimensional; model b: first-order with two correlated factors; model c: second-order hierarchical; model d: confirmatory bi-factor. χ2: Chi2; df: degrees of freedom; RMSEA: root mean square error of approximation; CFI: comparative fit index; TLI: Tucker-Lewis Index; AIC: Akaike information criterion; ECVI: expected cross-validation index.

#### Temporal stability

The DMS-FR demonstrated adequate test–retest reliability. The Pearson product-moment correlation coefficient between the two sets of total scores (Time 1 and Time 2) was *r* = .86, *p* < .01, and for Muscularity Body Dissatisfaction, and Muscularity Behaviors, the correlation coefficients were .83, *p* < .01 and *r* = .86, *p* < .01, respectively. Estimates of internal consistency (Cronbach's alpha) in Time 2 were .81 and .74 for Muscularity Body Dissatisfaction and Muscularity Behaviors, respectively.

#### Concurrent validity

Concurrent validity was examined by testing the relationships between the DMS-FR and the MBDS. The total score of the DMS-FR was positively related to the MBDS: *r* = .35, *p* < .01. The two subscales of the DMS-FR (Muscularity Body Dissatisfaction and Muscularity Behaviors) were significantly and positively correlated with the Muscularity Body Dissatisfaction subscale of the MBDS (*r* = .46; *p* < .01 and *r* = .34; *p* < .01, respectively). The Muscularity Body Dissatisfaction subscale of the DMS-FR and the General Body Appearance Dissatisfaction subscale of the MBDS were negatively related in the expected directions (*r* = -.28; *p* < .01). However, the General Body Appearance Dissatisfaction subscale of the MBDS and the Muscularity Behaviors subscale of the DMS were not significantly related (Table 4).

**Table 4:** Concurrent validity: Correlations between the DMS-FR and the subscales of the MBDS.

Correlations	*DMS* Muscularity Body Dissatisfaction	*DMS* Muscularity Behaviors	*DMS* Global
**MBDS. Muscularity Body Dissatisfaction**	.46**	.34**	.46**
**MBDS. General Body Appearance Dissatisfaction**	-.28**	.09	-.12
**MBDS Global**	.25**	.37**	.35**

**p < 0.01

### Discussion

The aims of study 2 were to evaluate the psychometric properties of the DMS-FR by a series of structural hypothetical modelisation according to the four steps of Myers et al. [40] and to test the temporal stability, internal consistency and concurrent validity of the instrument. The negative relation between the Muscularity Body Dissatisfaction subscale of the DMS-FR and the General Body Appearance Dissatisfaction subscale of the MBDS is unsurprising because the two subscales do not explain the same concept: Muscularity Body Dissatisfaction subscale concerns body dissatisfaction, while the General Body Appearance Dissatisfaction subscale concerns body satisfaction: these subscales are opposite.

First, the series of structural hypothetical modelisation showed that the most satisfactory model was the bi-factor model, with a general factor: Drive for Muscularity and two sub-factors: Muscularity Body Dissatisfaction and Muscularity Behaviors. Second, the DMS-FR demonstrated good test-retest reliability and internal consistency. Last, the associations between the DMS-FR and the MBDS were globally in the expected directions [20,53], thus providing the first support for its concurrent validity.

## General discussion

The purpose of this study was to adapt and validate a French short version of the Drive for Muscularity scale (DMS-FR) with a view to establishing a more theoretically based scale for future research. Two studies were carried out following the validation procedure of Vallerand [39]. The validity of the tool was successively demonstrated by PCA (Study 1) and the series of structural hypothetical modelisation (Study 2). The reliability of the DMS-FR was demonstrated by satisfactory temporal stability and good concurrent validity (Study 2). The factorial analysis retained a bi-factor model with Muscularity Body Dissatisfaction comprising four items, Muscularity Behaviors comprising five items, and one general factor of Drive for Muscularity on all nine items.

Contrary to studies where participants’ age had a significant effect on the overall DMS and younger men are more likely to desire a more muscular body [30], age did not have any effect in our study. Moreover, although previous studies [29] have shown that frequent weight-training session per week and exercise habits were related to high scores of the DMS, no significant effect of sport experience was found in our study.

Our results indicated a two-factor structure that mirrored the model proposed by McCreary et al. [54] and agreed with previous reports of its adequate-to-good fit in Scottish [35], Brazilian [29], Mexican [33], German [34], Argentine [32], and Malay male samples [27]. Nevertheless, some differences emerged. Earlier studies generally differentiated a Muscularity Attitudes subscale composed of items referring to various theoretical constructs (e.g., beliefs; body dissatisfaction; motivation) from a Muscularity Behaviors subscale. For better conceptual clarity, the DMS-FR was conceptualized on two dimensions, Muscularity Body Dissatisfaction and Muscularity Behaviors. The validation of a bi-factor model has the advantage of being able to determine the relative and joint influence of the total score and the subscales [55]. These results suggest that it will be possible to consider, in future studies, both general score (i.e., drive for muscularity), and specific scores of each subscale (i.e., Muscularity Body Dissatisfaction and Muscularity Behaviors). Given that the attitudinal and behavioral components of the DMS have been shown to have different associations with behavioral outcomes [56], the use of the two subscales will be useful to gain a better understanding of how the muscularity body dissatisfaction and muscularity behaviors of French males are related to other theoretically relevant constructs.

The DMS has been previously used in many populations: males [27], male adolescents [57], male students [20], male athletes [58], male and female students [19], homosexual and heterosexual men [30], and homosexual men [31]. Our results showed that, in line with the findings of previous studies in other countries, the DMS-FR subscales had satisfactory test-retest reliability across four weeks and good internal consistency [35]. The subscales of the DMS-FR were also significantly related to the Muscularity Body Dissatisfaction subscale of the MBDS [45], both questionnaires being expected to measure proximal constructs. Moreover, several studies have reported consistent relationships between body dissatisfaction and drive for muscularity [7].

This study presents some limitations and points to directions for future research. First, the data of studies 1 and 2 were collected online, which may have limited participation to those with access to a computer, smartphone, and/or stable internet connection. Second, a typical limitation of research based on self-report measures is the potential for social desirability bias. Third, our sample included only male athletes, and it is thus now necessary to test this survey in non-athlete males to generalize our results. An interesting question is whether the DMS scores are multi- or one-dimensional. Only two previous transcultural studies have examined fit of the higher-order dimensionality of DMS scores and have found equivocal results. Nerini et al. [30] reported that the higher-order dimensionality had adequate fit in Italian men, while Sepúlveda et al. [26] found that a model that included the higher-order factor had poor fit in Spanish adolescents. This calls for further investigation. Given that previous studies have reported significant relationships between the DMS and constructs like social physique anxiety [59], depression [60], and eating behaviors [61], future studies could confirm these associations with the DMS-FR in French-speaking samples. Future research could also examine the DMS in relation to other tools, such as the Muscle Dysmorphia Inventory (MDI) [24] or the Bodybuilding Dependence Scale (BDS) [62]. More generally, the adaptation and validation of the DMS-FR will be useful to conduct studies targeting French-speaking male athletes who are characterized by body dissatisfaction, intent on increasing muscle mass, and therefore more likely to adopt deviant behaviors. This scale should encourage researchers to examine the antecedents, consequences, and correlates of DM in French-speaking male athletes. In addition, a more accurate measure of the DM construct in French males would allow cross-cultural studies.

The drive for muscularity has been linked to significant physical and psychological problems in males [57]. The availability of this instrument in the French-language will therefore allow researchers, mental health professionals and educators to evaluate DM in French-speaking males and to intervene as needed with an appropriate preventive approach. With respect to practical implications, this scale could be administered for early identification of risk factors that might lead to dysfunctional behavior. We expect that the DMS-FR will also prompt more systematic investigation of men’s body image concerns in the French-speaking population.

## Conclusion

To conclude, the drive for muscularity is an important component of men's self-image. Theorists and researchers are currently at the beginning of the long path that will lead to a better understanding of the individual differences in men's body image perceptions and body-related behaviors. The DMS-FR can be used to measure the drive for muscularity in French-speaking male athletes and this should facilitate future research on the antecedents, consequences, and correlates of DM in this population. Greater insight into the drive for muscularity should also aid in the development of better prevention strategies for coaches and male athletes.

## Supporting information

S1 Dataset Data sample among participants of studies 1 and 2. Data used for the realization of the different analyses.
(XLSX)

## References

[1] Pope HG, Olivardia R, Gruber A, Borowiecki J. Evolving ideals of male body image as seen through action toys. International journal of eating disorders. 1999;26(1):65–72. 10349585 10.1002/(sici)1098-108x(199907)26:1<65::aid-eat8>3.0.co;2-d

[2] Baghurst T, Kissinger DB. Perspectives on muscle dysmorphia. International Journal of Men's Health. 2009;8(1):82 doi: 10.3149/jmh.0801.82

[3] Edwards C, Tod D, Molnar G. A systematic review of the drive for muscularity research area. International Review of Sport and Exercise Psychology. 2014;7(1):18–41. doi: 10.1080/1750984X.2013.847113

[4] Karazsia BT, Murnen SK, Tylka TL. Is body dissatisfaction changing across time? A cross-temporal meta-analysis. Psychological bulletin. 2017;143(3):293–320. doi: 10.1037/bul0000081 27893220 10.1037/bul0000081

[5] Labre MP. The male body ideal: Perspectives of readers and non-readers of fitness magazines. The journal of men's health & gender. 2005;2(2):223–9. doi: 10.1016/j.jmhg.2005.03.001

[6] Wyssen A, Bryjova J, Meyer AH, Munsch S. A model of disturbed eating behavior in men: The role of body dissatisfaction, emotion dysregulation and cognitive distortions. Psychiatry research. 2016;246:9–15. doi: 10.1016/j.psychres.2016.09.010 27639817 10.1016/j.psychres.2016.09.010

[7] Olivardia R, Pope HG Jr, Borowiecki JJ III, Cohane GH. Biceps and Body Image: The Relationship Between Muscularity and Self-Esteem, Depression, and Eating Disorder Symptoms. Psychology of men & masculinity. 2004;5(2):112–20.

[8] Smolak L, Stein JA. The relationship of drive for muscularity to sociocultural factors, self-esteem, physical attributes gender role, and social comparison in middle school boys. Body image. 2006;3(2):121–9. doi: 10.1016/j.bodyim.2006.03.002 18089215 10.1016/j.bodyim.2006.03.002

[9] Babusa B, Túry F. Muscle dysmorphia in Hungarian non-competitive male bodybuilders. Eating and Weight Disorders-Studies on Anorexia, Bulimia and Obesity. 2012;17(1):e49–e53. doi: 10.1007/BF03325327 10.1007/BF03325327 22751271

[10] Pope HG Jr, Gruber AJ, Choi P, Olivardia R, Phillips KA. Muscle Dysmorphia: An Underrecognized Form of Body Dysmorphic Disorder. Psychosomatics. 1997;38(6):548–57. doi: 10.1016/S0033-3182(97)71400-2 9427852 10.1016/S0033-3182(97)71400-2

[11] Pope HG Jr, Katz DL, Hudson JI. Anorexia nervosa and “reverse anorexia” among 108 male bodybuilders. Comprehensive Psychiatry. 1993;34(6):406–9. doi: 10.1016/0010-440X(93)90066-D 8131385 10.1016/0010-440x(93)90066-d

[12] Contesini N, Adami F, Blake Md-T, Monteiro CB, Abreu LC, Valenti VE, et al Nutritional strategies of physically active subjects with muscle dysmorphia. International archives of medicine. 2013;6(1):25 doi: 10.1186/1755-7682-6-25 23706013 10.1186/1755-7682-6-25 PMC3680023

[13] Mitchell L, Murray SB, Cobley S, Hackett D, Gifford J, Capling L, et al Muscle dysmorphia symptomatology and associated psychological features in bodybuilders and non-bodybuilder resistance trainers: A systematic review and meta-analysis. Sports Medicine. 2017;47(2):233–59. doi: 10.1007/s40279-016-0564-3 27245060 10.1007/s40279-016-0564-3

[14] Bahri A, Mahfouz MS, Marran NM, Dighriri YH, Alessa HS, Khwaji MO, et al Prevalence and awareness of anabolic androgenic steroid use among male body builders in Jazan, Saudi Arabia. Tropical Journal of Pharmaceutical Research. 2017;16(6):1425–30. doi: 10.4314/tjpr.v16i6.29

[15] García-Rodríguez J, Alvarez-Rayón G, Camacho-Ruíz J, Amaya-Hernández A, Mancilla-Díaz JM. Muscle dysmorphia and use of ergogenics substances. A systematic review. Revista Colombiana de Psiquiatría (English ed). 2017;46(3):168–77. doi: 10.1016/j.rcpeng.2017.06.003 10.1016/j.rcp.2016.06.008 28728801

[16] Olivardia R. Mirror, mirror on the wall, who's the largest of them all? The features and phenomenology of muscle dysmorphia. Harvard review of psychiatry. 2001;9(5):254–9. 11553529

[17] Maida DM, Armstrong SL. The classification of muscle dysmorphia. International Journal of Men's Health. 2005;4(1):73.

[18] Boyda D, Shevlin M. Childhood victimisation as a predictor of muscle dysmorphia in adult male bodybuilders. The Irish Journal of Psychology. 2011;32(3–4):105–15. doi: 10.1080/03033910.2011.616289

[19] McCreary DR, Sasse DK. An exploration of the drive for muscularity in adolescent boys and girls. Journal of American college health. 2000;48(6):297–304. doi: 10.1080/07448480009596271 10863873 10.1080/07448480009596271

[20] Morrison TG, Morrison MA, Hopkins C, Rowan ET. Muscle Mania: Development of a New Scale Examining the Drive for Muscularity in Canadian Males. Psychology of Men & Masculinity. 2004;5(1):30–9. doi: 10.1037/1524-9220.5.1.30

[21] Gillen M, Markey CN. Development and Validation of the Muscle Pictorial Measure. Archives of Assessment Psychology. 2015;5(1):11–22.

[22] Mayville SB, Williamson DA, White MA, Netemeyer RG, Drab DL. Development of the Muscle Appearance Satisfaction Scale: A self-report measure for the assessment of muscle dysmorphia symptoms. Assessment. 2002;9(4):351–60. doi: 10.1177/1073191102238156 12462755 10.1177/1073191102238156

[23] Ochner CN, Gray JA, Brickner K. The development and initial validation of a new measure of male body dissatisfaction. Eating behaviors. 2009;10(4):197–201. doi: 10.1016/j.eatbeh.2009.06.002 19778747 10.1016/j.eatbeh.2009.06.002

[24] Rhea D, Lantz CD, Cornelius AE. Development of the muscle dysmorphia inventory (MDI). Journal of Sports Medicine and Physical Fitness. 2004;44(4):428–35. 15758857

[25] McCreary DR, Sasse DK, Saucier DM, Dorsch KD. Measuring the Drive for Muscularity: Factorial Validity of the Drive for Muscularity Scale in Men and Women. Psychology of Men & Masculinity. 2004;5(1):49 doi: 10.1037/1524-9220.5.1.49

[26] Sepulveda AR, Parks M, de Pellegrin Y, Anastasiadou D, Blanco M. Validation of the Spanish version of the Drive for Muscularity Scale (DMS) among males: Confirmatory factor analysis. Eating behaviors. 2016;21(1):116–22. doi: 10.1016/j.eatbeh.2016.01.010 26829369 10.1016/j.eatbeh.2016.01.010

[27] Swami V, Barron D, Lau PL, Jaafar JL. Psychometric properties of the Drive for Muscularity Scale in Malay men. Body image. 2016;17(1):111–6. doi: 10.1016/j.bodyim.2016.03.004 27037873 10.1016/j.bodyim.2016.03.004

[28] Swami V, Vintila M, Tudorel O, Goian C, Barron D. Factor structure and psychometric properties of a Romanian translation of the drive for Muscularity Scale (DMS) in university men. Body image. 2018;25:48–55. doi: 10.1016/j.bodyim.2018.02.004 29475191 10.1016/j.bodyim.2018.02.004

[29] Campana ANNB, Tavares MdCGC, Swami V, da Silva D. An examination of the psychometric properties of Brazilian Portuguese translations of the Drive for Muscularity Scale, the Swansea Muscularity Attitudes Questionnaire, and the Masculine Body Ideal Distress Scale. Psychology of Men & Masculinity. 2013;14(4):376–88. doi: 10.1037/a0030087

[30] Nerini A, Matera C, Baroni D, Stefanile C. Drive for muscularity and sexual orientation: Psychometric properties of the Italian version of the Drive for Muscularity Scale (DMS) in straight and gay men. Psychology of Men & Masculinity. 2016;17(2):137–46. doi: 10.1037/a0039675

[31] DeBlaere C, Brewster ME. A confirmation of the Drive for Muscularity Scale with sexual minority men. Psychology of Sexual Orientation and Gender Diversity. 2017;4(2):227–32. doi: 10.1037/sgd0000224

[32] Compte EJ, Sepúlveda AR, de Pellegrin Y, Blanco M. Confirmatory factor analysis of the Drive for Muscularity Scale-S (DMS-S) and Male Body Attitudes Scale-S (MBAS-S) among male university students in Buenos Aires. Body image. 2015;14(1):13–9. doi: 10.1016/j.bodyim.2015.02.005 25828841 10.1016/j.bodyim.2015.02.005

[33] Escoto C, Alvarez-Rayón G, Mancilla-Díaz JM, Ruiz EJC, Paredes KF, Lugo CSJ. Psychometric properties of the Drive for Muscularity Scale in Mexican males. Eating and Weight Disorders-Studies on Anorexia, Bulimia and Obesity. 2013;18(1):23–8. doi: 10.1007/s40519-013-0010-6 23757247 10.1007/s40519-013-0010-6

[34] Waldorf M, Cordes M, Vocks S, McCreary D. „Ich wünschte, ich wäre muskulöser”: Eine teststatistische Überprüfung der deutschsprachigen Fassung der Drive for Muscularity Scale (DMS). Diagnostica. 2014;60(1):140–52. doi: 10.1026/0012-1924/a000106

[35] McPherson KE, McCarthy P, McCreary DR, McMillan S. Psychometric evaluation of the Drive for Muscularity Scale in a community-based sample of Scottish men participating in an organized sporting event. Body Image. 2010;7(4):368–71. doi: 10.1016/j.bodyim.2010.06.001 20627231 10.1016/j.bodyim.2010.06.001

[36] Swami V, Diwell R, McCreary DR. Sexuality and the drive for muscularity: Evidence of associations among British men. Body image. 2014;11(4):543–6. doi: 10.1016/j.bodyim.2014.08.008 25201097 10.1016/j.bodyim.2014.08.008

[37] Tod D, Morrison TG, Edwards C. Psychometric properties of Yelland and Tiggemann's Drive for Muscularity Scale. Body Image. 2012;9(3):421–4. doi: 10.1016/j.bodyim.2012.03.003 22541667 10.1016/j.bodyim.2012.03.003

[38] Ajzen I. From intentions to actions: A theory of planned behavior Action control: Springer; 1985 p. 11–39.

[39] Vallerand RJ. Vers une méthodologie de validation trans-culturelle de questionnaires psychologiques: Implications pour la recherche en langue française. Canadian Psychology/Psychologie Canadienne. 1989;30(4):662–80. doi: 10.1037/h0079856 6850979

[40] Myers ND, Martin JJ, Ntoumanis N, Celimli S, Bartholomew KJ. Exploratory bifactor analysis in sport, exercise, and performance psychology: A substantive-methodological synergy. Sport, Exercise, and Performance Psychology. 2014;3(4):258–72. doi: 10.1037/spy0000015

[41] Beaton DE, Bombardier C, Guillemin F, Ferraz MB. Guidelines for the process of cross-cultural adaptation of self-report measures. Spine. 2000;25(24):3186–91. doi: 10.1097/00007632-200012150-00014 11124735 10.1097/00007632-200012150-00014

[42] O’connor BP. SPSS and SAS programs for determining the number of components using parallel analysis and Velicer’s MAP test. Behavior research methods, instruments, & computers. 2000;32(3):396–402. 10.3758/bf03200807 11029811

[43] Guttman L. Some necessary conditions for common-factor analysis. Psychometrika. 1954;19(2):149–61.

[44] Nunally JC. Psychometric theory (2 e edition) McGraw-Hill New York, NY 1978.

[45] Rousseau A, Denieul M, Lentillon-kaestner V, Valls M. French validation of the Male Body Dissatisfaction Scale in a sample of young men. Journal de thérapie comportementale et cognitive. 2014;24(3):122–9. doi: 10.1016/j.jtcc.2014.07.001

[46] Tabachnick B. Fidell. Using Multivariate Statistics. Needham Hills. CA: Allyn & Bacon; 2001.

[47] Guadagnoli E, Velicer WF. Relation to sample size to the stability of component patterns. Psychological bulletin. 1988;103(2):265 doi: 10.1037/0033-2909.103.2.265 3363047 10.1037/0033-2909.103.2.265

[48] MacCallum RC, Widaman KF, Preacher KJ, Hong S. Sample size in factor analysis: The role of model error. Multivariate Behavioral Research. 2001;36(4):611–37. doi: 10.1207/S15327906MBR3604_06 26822184 10.1207/S15327906MBR3604_06

[49] Amos AJ. 7.0 user's guide. Chicago: SPSS. 2006.

[50] Hu Lt, Bentler PM. Cutoff criteria for fit indexes in covariance structure analysis: Conventional criteria versus new alternatives. Structural equation modeling: a multidisciplinary journal. 1999;6(1):1–55. doi: 10.1080/10705519909540118

[51] Vandenberg RJ, Lance CE. A review and synthesis of the measurement invariance literature: Suggestions, practices, and recommendations for organizational research. Organizational research methods. 2000;3(1):4–70. doi: 10.1177/109442810031002

[52] Motl RW, Conroy DE. Validity and factorial invariance of the Social Physique Anxiety Scale. Medicine & Science in Sports & Exercise. 2000;32(5):1007–17. doi: 10.1097/00005768-200005000-00020 10795794 10.1097/00005768-200005000-00020

[53] Bratland-Sanda S, Sundgot-Borgen J. Symptoms of eating disorders, drive for muscularity and physical activity among Norwegian adolescents. European Eating Disorders Review. 2012;20(4):287–93. doi: 10.1002/erv.1156 21898700 10.1002/erv.1156

[54] McCreary DR, Saucier DM, Courtenay WH. The Drive for Muscularity and Masculinity: Testing the Associations Among Gender-Role Traits, Behaviors, Attitudes, and Conflict. Psychology of Men & Masculinity. 2005;6(2):83 doi: 10.1037/1524-9220.6.2.83

[55] Gorsuch RL. Factor analysis Handbook of psychology: Research methods in psychology. 2003;2:143–64.

[56] Litt D, Dodge T. A longitudinal investigation of the Drive for Muscularity Scale: Predicting use of performance enhancing substances and weightlifting among males. Body Image. 2008;5(4):346–51. doi: 10.1016/j.bodyim.2008.04.002 18644753 10.1016/j.bodyim.2008.04.002

[57] Pope H, Phillips KA, Olivardia R. The Adonis complex: The secret crisis of male body obsession: Simon and Schuster; 2000.

[58] de Sousa Fortes L, Ferreira MEC, de Oliveira SMF, Cyrino ES, Almeida SS. A socio-sports model of disordered eating among Brazilian male athletes. Appetite. 2015;92(1):29–35. doi: 10.1016/j.appet.2015.05.005 25963103 10.1016/j.appet.2015.05.005

[59] Martin KA, Rejeski WJ, Leary MR, McAuley E, Bane S. Is the Social Physique Anxiety Scale really multidimensional? Conceptual and statistical arguments for a unidimensional model. Journal of Sport and Exercise Psychology. 1997;19(4):359–67. doi: 10.1123/jsep.19.4.359

[60] Lovibond PF, Lovibond SH. The structure of negative emotional states: Comparison of the Depression Anxiety Stress Scales (DASS) with the Beck Depression and Anxiety Inventories. Behaviour research and therapy. 1995;33(3):335–43. doi: 10.1016/0005-7967(94)00075-U 7726811 10.1016/0005-7967(94)00075-u

[61] Garner DM, Garfinkel PE. The Eating Attitudes Test: An index of the symptoms of anorexia nervosa. Psychological medicine. 1979;9(2):273–9. doi: 10.1017/S0033291700030762 472072 10.1017/s0033291700030762

[62] Smith D, Hale B. Validity and factor structure of the bodybuilding dependence scale. British Journal of Sports Medicine. 2004;38(2):177–81. doi: 10.1136/bjsm.2002.003269 15039255 10.1136/bjsm.2002.003269 PMC1724773

